# KRT17 From Keratinocytes With High Glucose Stimulation Inhibit Dermal Fibroblasts Migration Through Integrin α11

**DOI:** 10.1210/jendso/bvad176

**Published:** 2024-01-02

**Authors:** Peng Zhou, Yiqing Li, Shan Zhang, Dian-Xi Chen, Ruikang Gao, Peiliang Qin, Chao Yang, Qin Li

**Affiliations:** Department of Vascular Surgery, Union Hospital, Tongji Medical College, Huazhong University of Science and Technology, Wuhan 430000, China; Department of Vascular Surgery, Union Hospital, Tongji Medical College, Huazhong University of Science and Technology, Wuhan 430000, China; Department of Vascular Surgery, Union Hospital, Tongji Medical College, Huazhong University of Science and Technology, Wuhan 430000, China; Department of Vascular Surgery, Union Hospital, Tongji Medical College, Huazhong University of Science and Technology, Wuhan 430000, China; Department of Vascular Surgery, Union Hospital, Tongji Medical College, Huazhong University of Science and Technology, Wuhan 430000, China; Department of Vascular Surgery, Union Hospital, Tongji Medical College, Huazhong University of Science and Technology, Wuhan 430000, China; Department of Vascular Surgery, Union Hospital, Tongji Medical College, Huazhong University of Science and Technology, Wuhan 430000, China; Department of Vascular Surgery, Union Hospital, Tongji Medical College, Huazhong University of Science and Technology, Wuhan 430000, China

**Keywords:** diabetes, KRT17, fibroblasts, proliferation, migration, ITGA11

## Abstract

**Objective:**

To investigate the effects of overexpressed keratin 17 (KRT17) on the biology of human dermal fibroblasts (HDFs) and to explore the mechanism of KRT17 in diabetic wound healing.

**Methods:**

KRT17 expression was tested in diabetic keratinocytes, animal models, and patient skin tissues (Huazhong University of Science and Technology Ethics Committee, [2022] No. 3110). Subsequently, HDFs were stimulated with different concentrations of KRT17 in vitro. Changes in the proliferation and migration of HDFs were observed. Then, identification of KRT17-induced changes in dermal fibroblast of RNA sequencing–based transcriptome analysis was performed.

**Results:**

KRT17 expression was upregulated under pathological conditions. In vitro stimulation of HDFs with different concentrations of KRT17 inhibited cell migration. RNA-seq data showed that enriched GO terms were extracellular matrix components and their regulation. KEGG analysis revealed that the highest number of enriched genes was PI3K-Akt, in which integrin alpha-11 (ITGA11) mRNA, a key molecule that regulates cell migration, was significantly downregulated. Decreased ITGA11 expression was observed after stimulation of HDFs with KRT17 in vitro.

**Conclusion:**

Increased expression of KRT17 in diabetic pathological surroundings inhibits fibroblast migration by downregulating the expression of ITGA11. Thus, KRT17 may be a molecular target for the treatment of diabetic wounds.

Diabetes mellitus is one of the most common systemic chronic metabolic diseases, mainly manifested by symptoms of hyperglycemia caused by defective insulin secretion and/or insulin function, and it is a serious threat to human health worldwide [1, 2]. Diabetes mellitus and its associated complications have a serious impact on the long-term life expectancy and quality of life of patients [3, 4]. Patients with diabetes are prone to skin lesions, such as pruritus, necrobiosis lipoidica, scleredema adultorum of Buschke, and granuloma annulare [5]. Apart from these noninfectious skin diseases, skin ulcers, and in severe cases, diabetic foot complications, endanger the limbs and lives of patients [6]. The treatment of diabetic skin ulcers and diabetic foot is a difficult clinical problem, and exploring the mechanisms underlying their occurrence and development is important.

Human skin comprises 3 layered structures: epidermis, dermis, and hypodermis. The integrity and stability of the dermis and epidermis are the basis for the protection of the skin against invasion by foreign harmful substances [7, 8]. The epidermis is mainly composed of keratinocytes, and the dermis is mainly composed of fibroblasts and microvascular endothelial cells. Studies have shown that the biological functions of skin keratinocytes, fibroblasts, and microvascular endothelial cells are altered by high glucose [9-11]. In our group, we performed transcriptome sequencing analysis (RNA-seq) of differential gene expression in skin keratinocytes, fibroblasts, and microvascular endothelial cells under high glucose conditions [12]. The expression was consistently upregulated in all 3 cell lines, suggesting that keratin 17 (KRT17) may play an important role in diabetic skin lesions.

KRT17 is an important member of the type I keratin family and is mainly expressed in basal cells of the epithelium, especially in keratinocytes [13]. Mutations in the KRT17 gene result in alterations in the structure of KRT17, which disrupts the integrity of the epidermis and can lead to the development of genetic disorders of the skin, such as congenital nail hypertrophy and multiple lipodystrophies. KRT17 acts as a cytoskeletal protein and can regulate a variety of biological processes, including skin cell proliferation and growth, skin inflammation, and follicular circulation [14]. Thus, KRT17 can be involved in a variety of diseases, including wound healing, activation of immune responses as an autoantigen in the development of psoriasis, and hair loss. As research continues, researchers have gradually discovered that KRT17 can be involved in regulating tumorigenesis and metastasis in epithelial cells and their derived cells [15, 16] and can also serve as a biomarker for predicting the prognosis of a variety of tumors [14]. Thus, it is clear that KRT17, although a skeletal protein, can play a variety of biological regulatory functions in a wide range of diseases, especially those involved in skin lesions. However, through an extensive review of the literature, no relevant studies on KRT17 in diabetic skin ulcers have been conducted.

Skin wound healing is a complex biological process involving various cells and cytokines [17]. There is increasing evidence that fibroblasts proliferate, migrate, and convert to myofibroblasts during wound healing, and secrete various cytokines that play an important role in wound healing [18-20]. In the early stages of injury, fibroblasts at the wound edge begin to proliferate and migrate into the fibrin clot of the wound, producing a large number of matrix proteins, such as collagen, proteoglycans, and elastin, which are involved in the formation of granulation tissue. Subsequently, fibroblasts migrate to the wound surface and gradually transform into a profibrotic phenotype that promotes protein synthesis. Alternatively, fibroblasts can be transformed into myofibroblasts, which are involved in trauma contraction [21].

Based on the group's previous findings on the upregulation of KRT17 mRNA in 3 types of skin cells (human epidermal keratinocyte [HEK], human dermal fibroblast [HDF], and human dermal microvascular endothelial cell [HDMEC]) under high glucose conditions, we first validated it in diabetic cell models, animal models, and patient skin tissues, and then established an in vitro KRT17-stimulated HDF cell model to explore the role of KRT17 in diabetic skin wound healing by observing the effects of KRT17 on HDF cell proliferation and migration.

## Methods

### Cell Cultures, Sample Preparations, and Reagents

The detailed steps for cell culture were in accordance with those described in our previous studies. Briefly, keratinocytes (HEK, Cat # 2110, ScienCell) were cultured in normal (6mM) and high (30mM) glucose media for 24 hours respectively, to simulate diabetic conditions. Similarly, the human immortalized keratinocyte (HaCaT) cell line (Cat #CL-0090, Procell) was cultured in MEM (Cat #PYG0029, Boster) and DMEM (Cat#DZPYG0209, Boster) medium for 24 hours respectively, to simulate diabetic conditions. Total RNA from the cell samples was extracted using RNAiso Plus reagent (Cat # 9108, Takara). Total protein was extracted from the cells using RIPA lysis buffer (Cat # P0013K, Beyotime) following the manufacturer's protocol. Supernatants were collected.

### Acquisition and Preparation of Skin Tissues

A total of 10 participants, comprising 5 subjects with concomitant diabetes mellitus and 5 control subjects without diabetes participated in this study. All of the included patients with diabetes mellitus had a history of chronic diabetes, with duration longer than 10 years. Skin tissues from patients with and without diabetes were collected during surgery. All of the participants provided written informed consent (Huazhong University of Science and Technology Ethics Committee, [2022] No. 3110). In addition, we created a type 2 diabetes mouse model and purchased db/db mice (Cat# HM0046, Shulb) to collect their skin tissues. This project was approved by the Wuhan Union Hospital Ethics Committee (S1983). A high-fat diet combined with streptozotocin injections was used to establish a diabetic mouse model, which is considered suitable for inducing the hallmark features of human type 2 diabetes [22]. Briefly, after fasting for 12 hours, streptozotocin (STZ; 120 mg/kg, Cat # S1312, Selleck) was administered intraperitoneally following a high-fat diet for 4 weeks. Glucose readings of nonfasted mice were recorded every 5 days. Thereafter, the mice were euthanized, and the dorsal skin was harvested for further analysis. All animals received care in compliance with the Principles of Animals Use Committee (NIH Publications No. 8023, revised 1978), and animals experiments adhere to the ARRIVE guidelines 2.0. The project was approved by the Wuhan Union Hospital Ethics Committee (REC 08/H1202/137), China.

### Quantitative Real-Time Reverse-Transcriptase Polymerase Chain Reaction

The cDNA was synthesized from RNA using a PrimeScript RT Reagent Kit (Cat # RR037A, Takara). SYBR Premix Ex Taq (Cat #RR420A, Takara) was used for quantitative polymerase chain reaction (qPCR) on an ABI Step One Plus System (Applied Biosystems, Foster City, CA, USA). The primers used were KRT17, 5′-CCCAGCTCAGCATGAAAGCA-3′ (forward), and 5′-ACAATGGTACGCACCTGACG3′ (reverse). All the primers were purchased from Sangon Biotech. The mRNA levels of the target genes were normalized to GAPDH (glyceraldehyde 3-phosphate dehydrogenase) using the 2−DDCT method.

### Western Blotting

Total proteins from HEK and HaCaT cells in 6-well plates and tissues were extracted using radioimmunoprecipitation assay (RIPA) lysis buffer containing 2% phenylmethylsulfonyl fluoride (PMSF) and phosphatase inhibitor (Cat # P0013K, Beyotime). Proteins were separated using 10% sodium dodecyl sulfate–polyacrylamide gel electrophoresis and transferred onto a nitrocellulose membrane. Protein probing was performed by overnight membrane incubation at 4 °C with primary antibodies (KRT17, Cat #18502-1-AP, Proteintech, RRID: AB 10644296). The membrane was then washed with TBST and incubated with horseradish peroxidase-conjugated secondary antibody (Cat# AS014, ABclonal, RRID:AB 2769854) for 2 hours. Blots were developed using enhanced chemiluminescence reagent (Cat # MA0186, Meilunbio), and band intensities were analyzed using ImageJ software (NIH, USA).

### Enzyme-Linked Immunosorbent Assay

The expression levels of KRT17 in the supernatant of HEK and HaCaT cells were measured using an enzyme-linked immunosorbent assay (ELISA) kit (Cat #, JL15259-48T, Jiang Lai Biotech) and a microplate reader (Thermo Fisher Scientific, USA). The ELISA was performed according to the manufacturer's instructions. The primary antibody of KRT17 in the kit was from Proteintech company (RRID: AB 10644296).

### Immunohistochemistry

All staining of skin samples was conducted by the Biossci Company (Hubei, China). The skin samples were fixed in 4% paraformaldehyde, washed in phosphate-buffered saline (PBS), dehydrated, and embedded in paraffin. Tissue sections were treated with primary antibodies (KRT17, Cat #18502-1-AP, Proteintech, RRID: AB 10644296), followed by incubation with the appropriate secondary antibodies (Cat# PR30009, Proteintech, RRID:AB 2934294). DAB (DAKO) was used to visualize the reaction, followed by counterstaining with hematoxylin. The sections were then analyzed for red color using a light microscope.

### Stimulation of Recombinant Human Cytokeratin 17

HDFs (Cat # 2320, ScienCell) were cultured in normal glucose (8 mM) and seeded in 6-well plates. At 60% to 70% confluence, the cells were starved in serum-free medium for 12 hours before stimulation with 0.1, 1, and 10 ng/mL recombinant human cytokeratin 17 (KRT17, Cat#PRO-1883, ProSpec).

### Observation of Cell Morphology and Growth

The growth of cells stimulated with different concentrations of KRT17 was observed and recorded daily under an inverted microscope.

### Cell Proliferation Assay

Cell proliferation was assessed using the Cell Counting Kit-8 (CCK8, Cat #CK04-500 T, Dojindo). HDF were seeded in 96-well plates at a density of 1 × 10^4^ cells/well before the replacement of fresh media containing different concentrations of KRT17 (0, 0.1, 1, and 10 ng/mL). After 24 hours, 48 hours, and 72 hours, cell proliferation was measured using the CCK-8 assay. Briefly, a total of 10 µL CCK-8 solution was added to each well containing 100 µL of culture medium and incubated for 1 hour at 37 °C. Finally, absorbance was measured at 450 nm using a microplate reader (Thermo Fisher, USA).

### Cell Migration Assay

Cell migration was assessed by scratch and Transwell assays. HDF cells were seeded in 3 12-well plates for each treatment. When the cell confluency reached 90% to 95%, 3 different scratches were made in each well. A scratch was made using a 20-μL pipette tip placed along the diameter of the well. Then, cells were starved in serum-free culture medium for 24 hours. Cells were washed with PBS to remove the scratched cells. Fresh complete culture medium containing different concentrations of KRT17 (0 and 1 ng/mL KRT17) was added. The images were then acquired from the same area for each treatment condition at 0, 12, and 24 hours. For the Transwell assay, 24-well Transwell chambers (Corning) were used. HDF cells were seeded into the upper layer in basal medium without fetal bovine serum (FBS) at a density of 1 × 10^5^ cells/well while the lower chamber was filled with different concentrations of KRT17 FM containing 10% FBS. Invading cells were fixed and quantified after 24 hours of incubation.

### RNA Extraction and RNA Sequencing Procedures

RNAs were extracted from cultivated HDF cells using RNAiso Plus (Cat # 9108, TaKaRa). RNA sequencing (RNA-seq) and RNA-seq analyses were performed using commercially available service (service ID # F21FTSCCWGT0114, BGI, Wuhan, China). Briefly, total RNA was extracted and mRNA was enriched using oligo (dT) beads for library construction. After library construction, a qualified library was selected for sequencing. Following sequencing of each cDNA library, the raw sequencing data were transformed into the original sequence data, termed raw data or raw reads. Raw sequencing read QC and filtering were performed using Fastp. After filtering, clean reads were aligned against the reference genome. Clean reads were processed using downstream analysis, including gene expression and deep analysis based on gene expression. The sequencing data that support the findings of this study have been deposited into CNGB Sequence Archive (CNSA) of China National GeneBank DataBase (CNGBdb) with accession number CNP0004789.

### Functional Enrichment Analysis

Normalization and differential expression analyses were estimated from count data using the DEGseq package in the analysis system of Dr. Tom from BGI. Differentially expressed genes (DEGs) were screened using FDR-adjusted q values (q values ≤ 0.05), and fold changes ≥1.2. DEGs were extracted for GO functional enrichment analysis and KEGG pathway enrichment analysis using the analysis system of Dr. Tom from the BGI. Data visualization was performed using a bubble diagram.

### Immunofluorescence Staining

HDF were seeded on circular coverslip slides in 24-well plates at a density of 5000 cells/well and cultured. After treatment with 1 ng/mL KRT17 complete medium for 24 hours, HDF were washed twice with PBS, fixed with 4% paraformaldehyde for 30 minutes, and rinsed twice with PBS. The cells were then treated with 0.1% Triton-X 100 for 10 minutes. Slides were blocked with 5% goat serum (Boster, Wuhan, China) for 1 hour, and incubated with integrin alpha 11 (ITGA11) primary antibodies (1:50, Cat # A10084, ABclonal Technology, RRID: AB 2757608) overnight at 4 °C. HaCaT cells were incubated with secondary antibodies (Cat# SA00003-2, Proteintech, RRID:AB 2890897) labeled with FITC (Green) (1:200, Servicebio) at 37 °C for 1 hour in the dark. The cells were subsequently stained with DAPI (blue) for 5 minutes in the dark. Images were acquired using a fluorescence microscope (Bio-Rad Laboratories).

### Statistical Analyses

Data are expressed as the mean ± SEM. Parametric and non-parametric quantitative variables were compared using the Student *t* test and Mann–Whitney U-test, respectively. The least significant difference (LSD) method in one-way ANOVA was used for pairwise comparisons between different groups. Statistical significance was set at *P* < .05. All figures were generated using GraphPad Prism 9.0 and Adobe Illustrator CC 2015.

## Results

### Increased KRT17 Expression in HEK and HaCaT Cultures Under High Glucose

**Figure 1.**
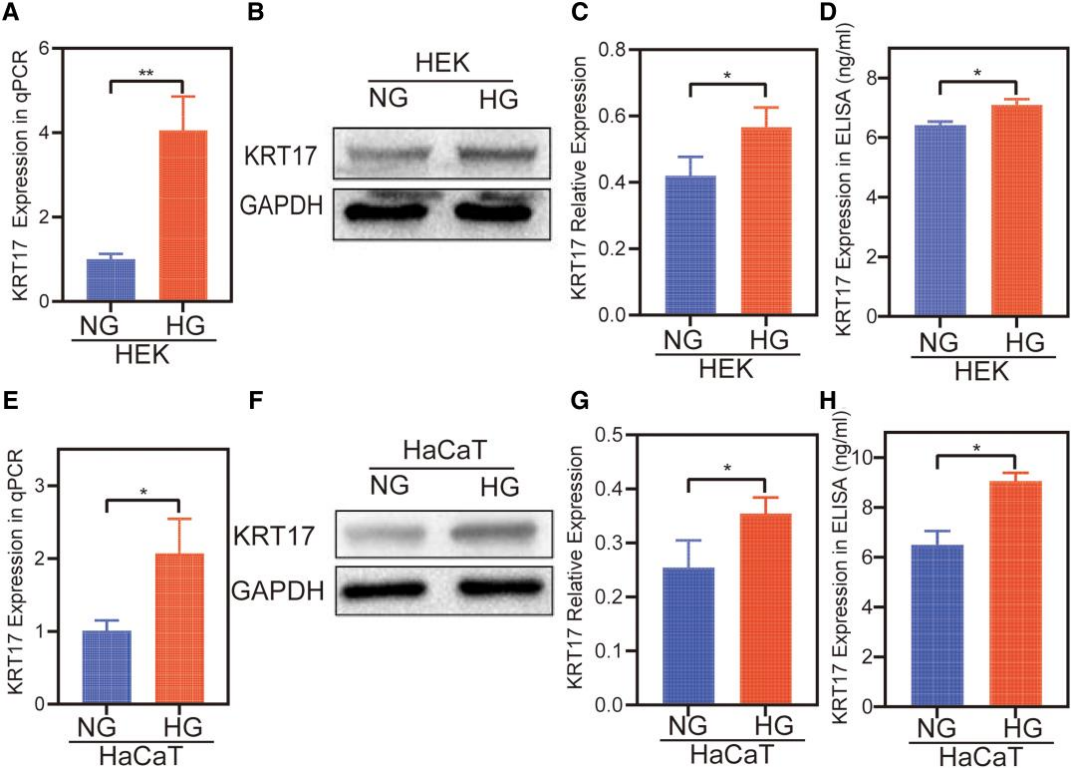
The expression of KRT17 in high glucose–treated HEKs and HaCaTs. (A-D) The validation of the expression of KRT17 in high glucose–treated HEKs with tests of RT-qPCR, Western blot, and ELISA methods. (E-H) The expression of KRT17 in high glucose–treated HaCaTs using the same methods. The independent experiment was repeated 3 times. The results are provided as the means ± SEM, *P < .05 compared with the control.

Although KRT17 mRNA expression was increased in our previous study using RNA sequencing–based transcriptome analysis, measuring gene expression in normal and high glucose–stimulated skin cells, the activity of KRT17 was indeterminate. We further investigated the changes in the protein expression of KRT17 using 2 cell lineages under high conditions. In the high glucose–stimulated HEK cell model, qPCR results showed increased expression of KRT17 mRNA (Fig. 1A), and Western blot results showed that KRT17 protein levels were higher (Fig. 1B and 1C), and the protein expression levels of KRT17 in the supernatant were increased (Fig. 1D). The same test showed increased KRT17 expression in the HG-stimulated HaCaT cell model (Fig. 1E-1H).

### Assessment of Diabetic Animal Models

In this study, 5 of the 7 mice were successfully established as diabetes models, defined as having glucose levels above 14 mmol/L (20, 21). The last measurement of plasma glucose concentration was used to calculate the average value (21.7 [19.8-22.3] mmol/L) for these diabetic mice.

### Increased KRT17 Expression in Diabetic Mouse Skin

**Figure 2.**
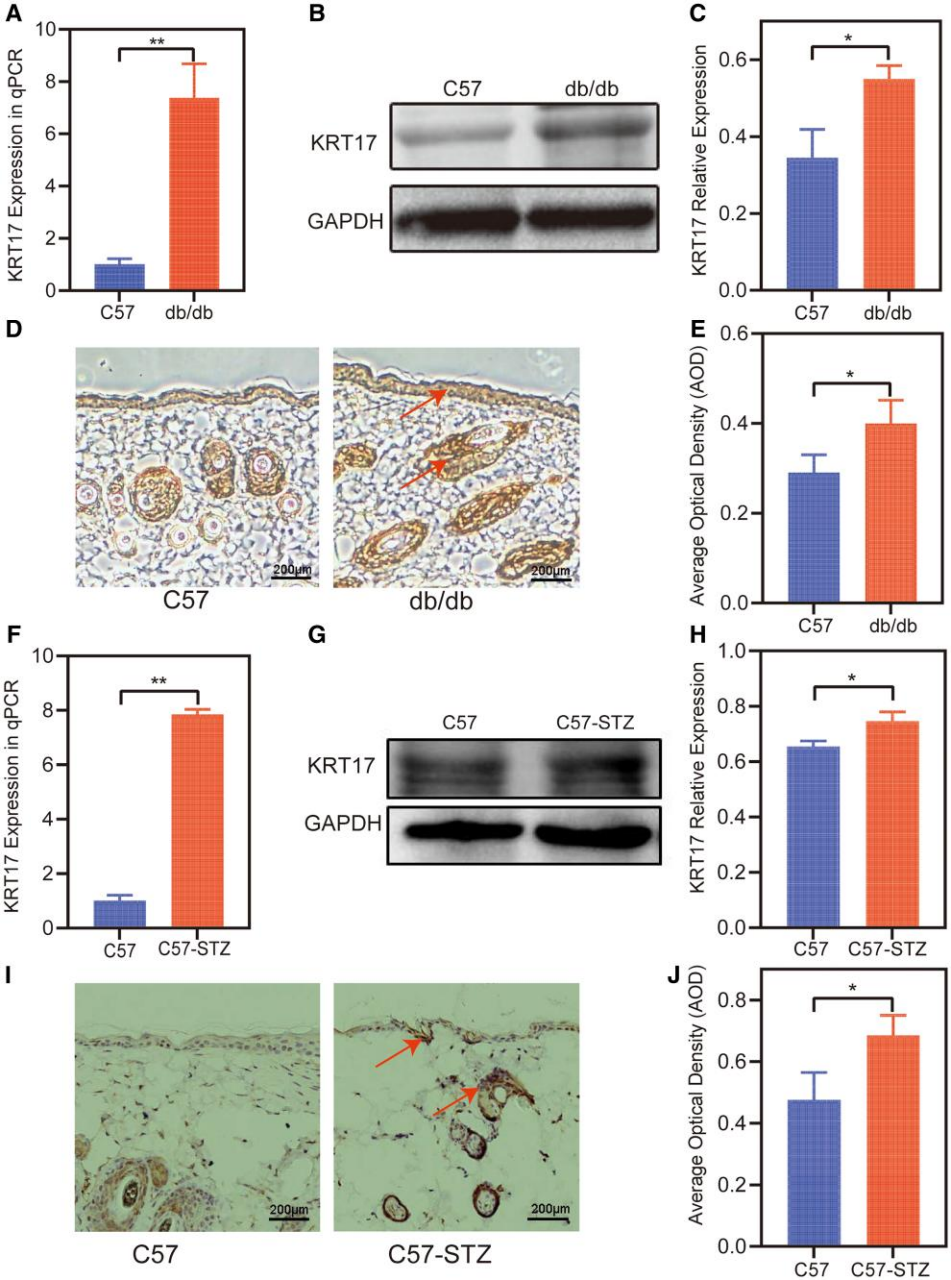
The expression of KRT17 in the skin of diabetic mouse. (A-E) The validation of the expression of KRT17 in the skin of db/db mouse with tests of RT-qPCR, Western blot, and immunohistochemical methods. (F-J) The expression of KRT17 in the skin of HFD/STZ treated mouse. The independent experiment was repeated 3 times. The results are provided as the means ± SEM, *P < .05 compared with the control.

To further elucidate the relationship between KRT17 and diabetes status, we investigated the expression of KRT17 in the skin tissue of diabetic mice. In the skin tissue of db/db diabetic mice, the qPCR results showed increased expression of KRT17 mRNA compared to that in the normal group (Fig. 2A). The Western blot results showed that KRT17 protein levels were higher (Fig. 2B and 2C), and the immunohistochemistry results showed that KRT17 was increased (Fig. 2D-2E). The same test showed increased KRT17 expression in the diabetic mouse model, high-fat diet combined with streptozotocin (HFD/STZ) (Fig. 2F-2J).

### Increased KRT17 Expression in Skin of Patients With Diabetes

**Figure 3.**
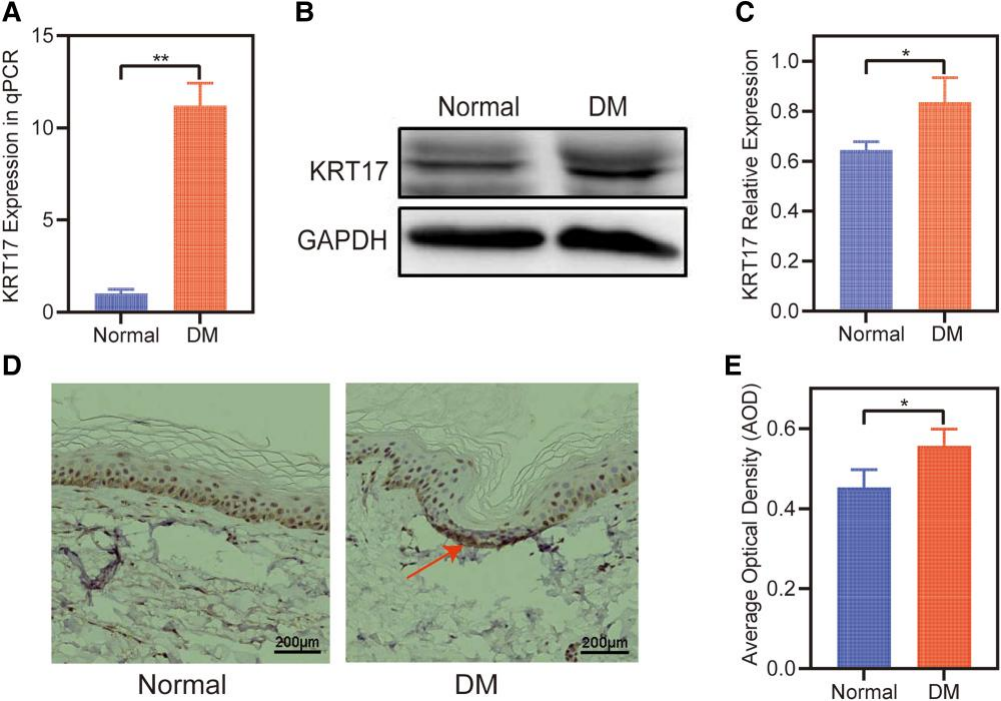
The expression of KRT17 in the skin of diabetics. (A) RT-qPCR analysis of KRT17 expression in the skin of diabetics compared with normal. (B and C) Western blots and quantitative analysis of KRT17 expression. (D and E) Immunohistochemical and quantitative analysis of KRT17 expression. The independent experiment was repeated 3 times. The results are provided as the means ± SEM, *P < .05 compared with the control.

Furthermore, we tested the expression of KRT17 in the skin of patients with diabetes. The qPCR results showed that the expression of KRT17 mRNA was increased in the control skin (Fig. 3A). Increased KRT17 protein expression was observed in skin of patients with diabetes by Western blotting (Fig. 3B and 3C) and immunohistochemistry (Fig. 3D and 3E) tests.

### Proliferation and Migration Effects of KRT17 on Dermal Fibroblasts

**Figure 4.**
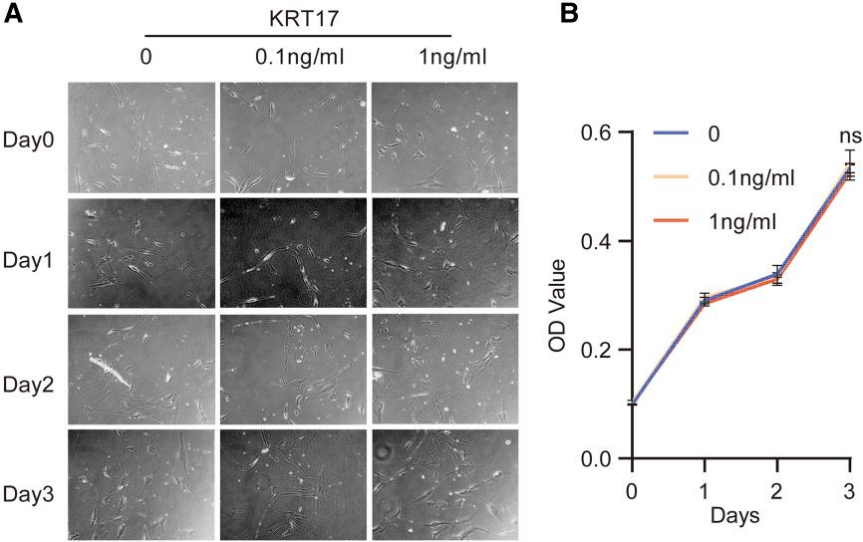
The effects of KRT17 on the proliferation of HDFs. (A) Cell morphological changes and high-density growth under a microscope. (B) A graph of the CCK8 assay results. The changes in the stimulation of HDFs with different concentration of KRT17 have no statistical difference. The independent experiment was repeated 3 times. The results are provided as the means ± SEM, *P < .05 compared with the control.

**Figure 5.**
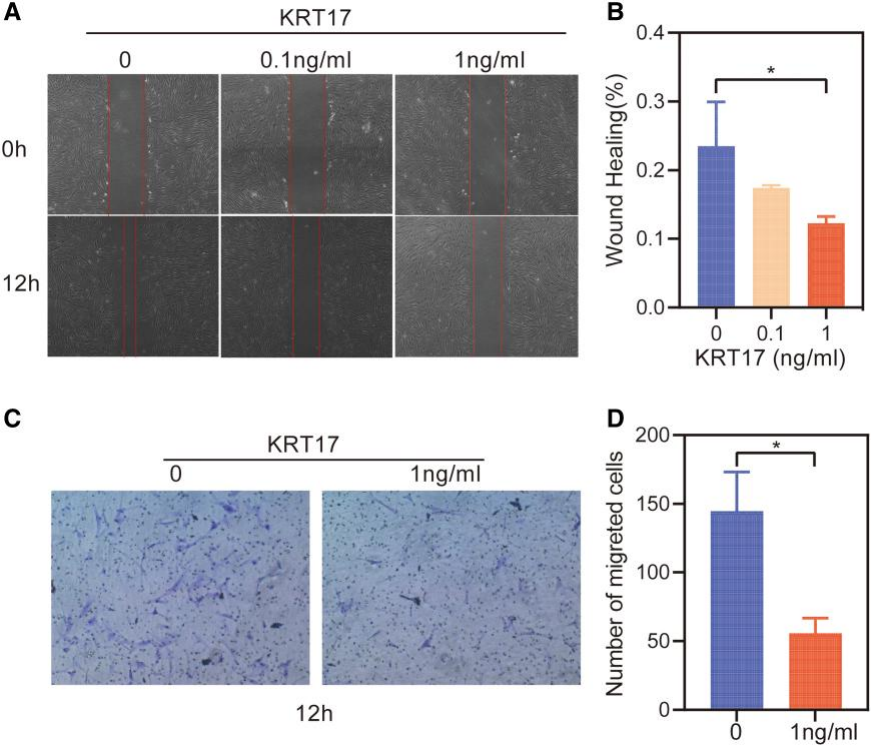
The effects of KRT17 on the cell migration of HDFs. (A) Results of cell scratching wound healing assay. (B) Statistical analysis of cell migration in the scratch wound healing assays. (C) Transwell invasion assay results. (D) Statistical analysis of the results from the Transwell migration assays. The independent experiment was repeated 3 times. The results are provided as the means ± SEM, *P < .05 compared with the control.

To clarify the effect of KRT17 on skin, we designed an in vitro stimulation experiment of HDF by KRT17 to explore its effect on dermal function. To investigate the effect of KRT17 on HDF proliferation, the culture was stimulated with 0.1 and 1 ng/mL KRT17. Microscopic observations indicated that the growth of HDF was not significantly different (Fig. 4A). The CCK8 assay revealed that stimulation with KRT17 had no effect on HDF cell proliferation (Fig. 4B). Further, HDF cell migration in response to KRT17 was measured using scratch wound and Transwell migration assays. Scratch wound assay showed that KRT17 inhibited HDF cell migration (Fig. 5A and 5B). Similarly, the results of the Transwell assay demonstrated that KRT17 stimulation significantly inhibited the migration ability of HDF (Fig. 5C and 5D).

### Identification of KRT17-Induced Changes in Dermal Fibroblasts by RNA Sequencing–Based Transcriptome Analysis

**Figure 6.**
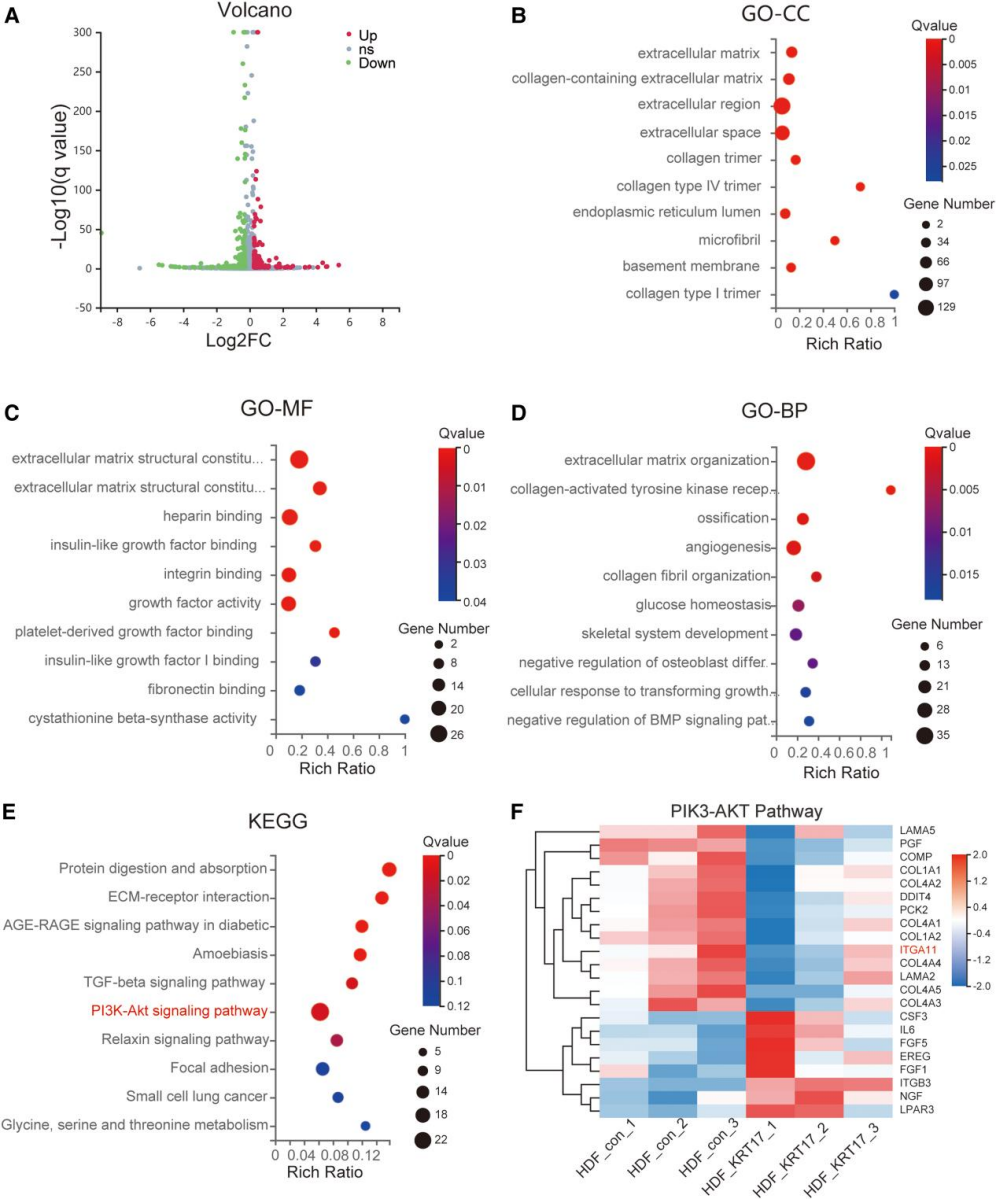
Bioinformatics analysis of DEGs in HDF stimulated with KRT17. (A) The volcano plot of differentially expressed genes. (B-D) Top 10 GO terms of cellular component (CC), molecular function (MF), and biological process (BP) are shown respectively. (E) Top 10 KEGG pathways of KEGG enrichment analyses of differentially expressed genes. (F) The heatmap of the differential gene expression analysis based on the enriched PI3K/AKT signaling pathway. The downregulated gene ITGA11 that suggested involved in cell migration is marked.

To further assess the role of KRT17 in HDF, we performed RNA sequencing–based transcriptome analysis by measuring gene expression patterns in biological replicates. A total of 537 DEGs were screened from common 15378 genes, including 293 downregulated and 244 upregulated genes. Data were visualized using a volcano plot (Fig. 6A). GO analysis of DEGs was conducted according to 3 GO categories: biological processes (BP), molecular functions (MF), and cellular components (CC). The top 10 enriched GO terms in each GO category are shown (Fig. 6B-6D). The significantly enriched GO terms were extracellular matrix components and their regulation. The top 10 significant KEGG pathways ranked by gene count are shown (Fig. 6E). The pathway enriched with the highest number of genes was the PI3K-Akt signaling pathway. As the PI3K-AKT pathway is a crucial signaling pathway in cellular processes, such as proliferation and migration, the 22 target genes in the PI3K/AKT pathway were analyzed in detail (Fig. 6F). Eight genes were upregulated and 14 were downregulated. Of the downregulated genes, ITGA11 was a transmembrane glycoproteins that function in cell adhesion and transduction of signals involved in cell migration.

### ITGA11 Expression Levels Were Decreased in HDF Cells After KRT17 Stimulation

**Figure 7.**
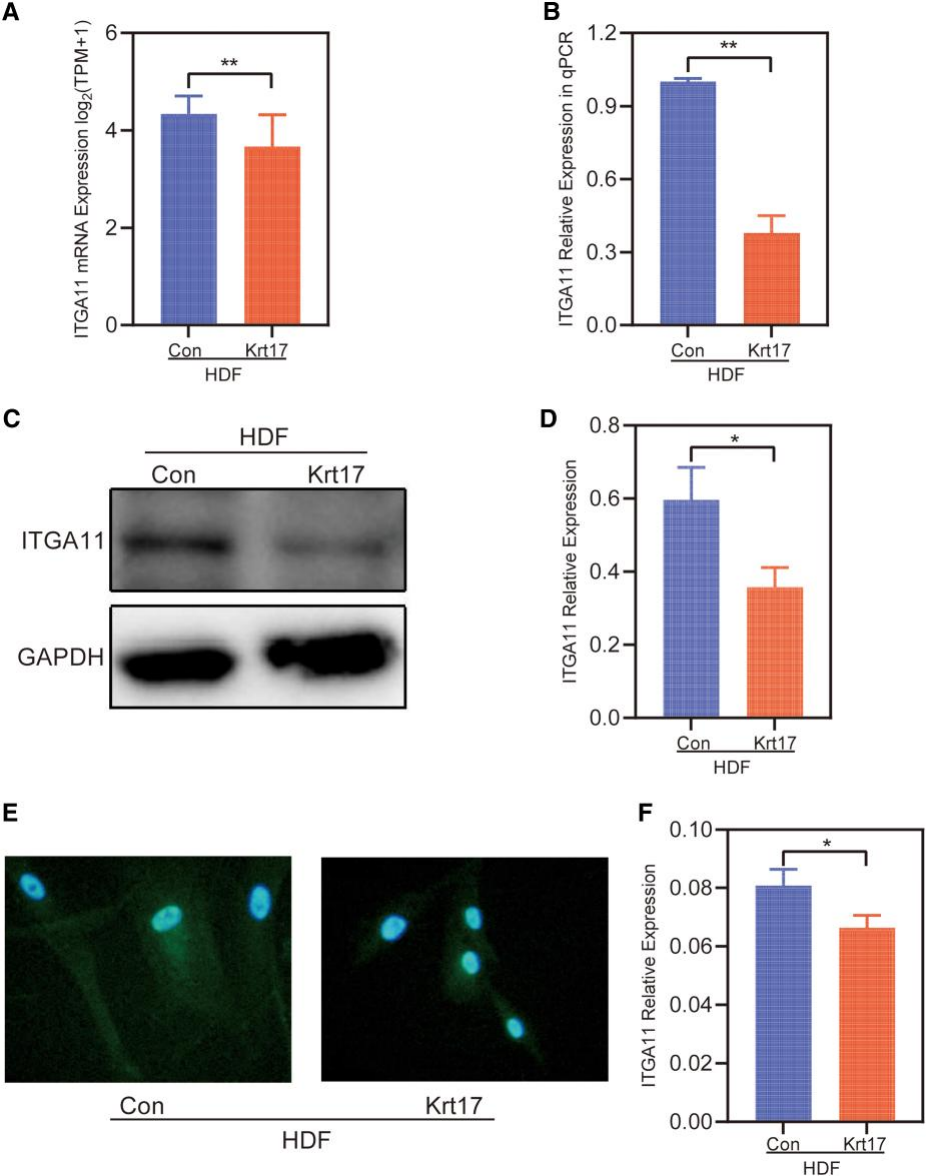
The expression of ITGA11 in HDF stimulated with KRT17. (A) RNA-seq quantification of the expression of ITGA11. (B) RT-qPCR validation of the expression of ITGA11. (C and D) Western blots and quantitative analysis of ITGA11. (E and F) ITGA11 Immunofluorescence and quantitative analysis. The independent experiment was repeated 3 times. The results are provided as the means ± SEM, *P < .05 compared with the control.

Given the role of ITGA11 in cell migration, we next performed experiments to verify whether KRT17 stimulation alters the expression of ITGA11. In RNA-seq, decreased ITGA11 mRNA expression levels were observed in HDF following KRT17 stimulation (Fig. 7A). Consistent with the RNA-seq results, the qPCR results showed that ITGA11 expression was decreased (Fig. 7B). To further validate the above results, Western blotting and immunofluorescence staining were performed, showing decreased ITGA11 protein expression levels (Fig. 7C-7F).

### Restoring ITGA11 Levels in the Presence of KRT17 Reversed the Cell Migration

**Figure 8.**
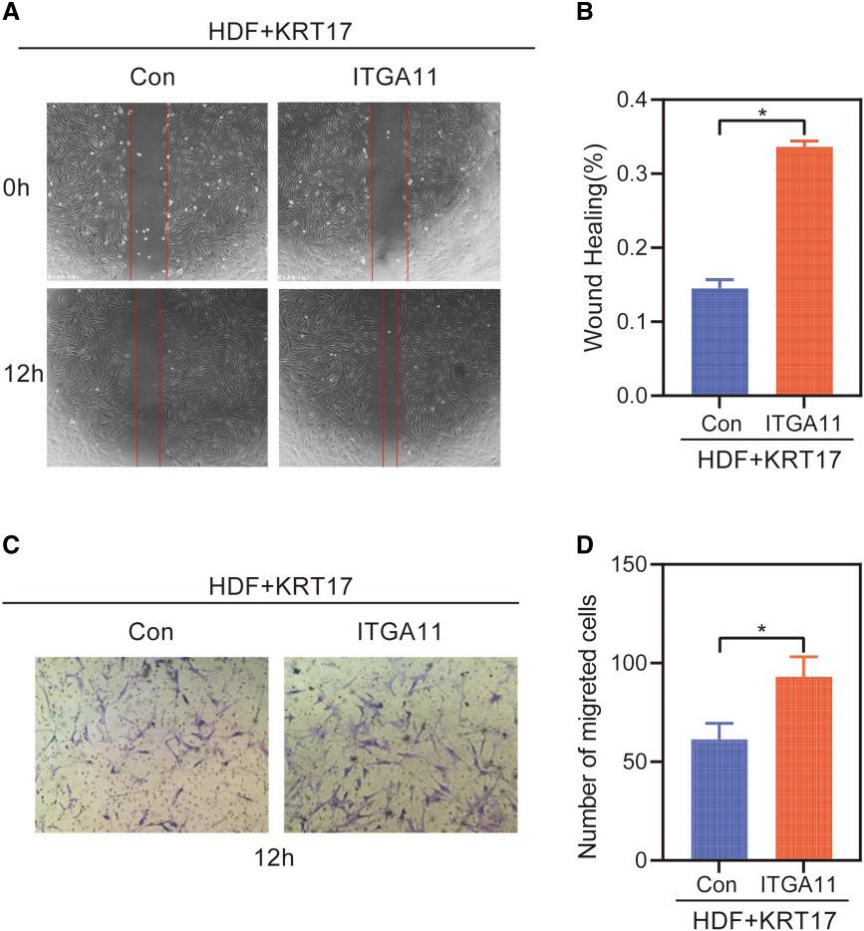
The migration effect of restoring ITGA11 levels in the presence of KRT17. (A) Results of cell scratching wound healing assay. (B) Statistical analysis of cell migration in the scratch wound healing assays. (C) Transwell invasion assay results. (D) Statistical analysis of the results from the Transwell migration assays. The independent experiment was repeated 3 times. The results are provided as the means ± SEM, *P < .05 compared with the control.

The migration effect of restoring ITGA11 levels in the presence of KRT17 is assessed. Scratch wound assay showed that ITGA11 reversed HDF cell migration by KRT17 (Fig. 8A and 8B). Similarly, the results of the Transwell assay demonstrated that ITGA11 stimulation significantly reversed the migration ability of HDF (Fig. 8C and 8D).

## Discussion

**Figure 9.**
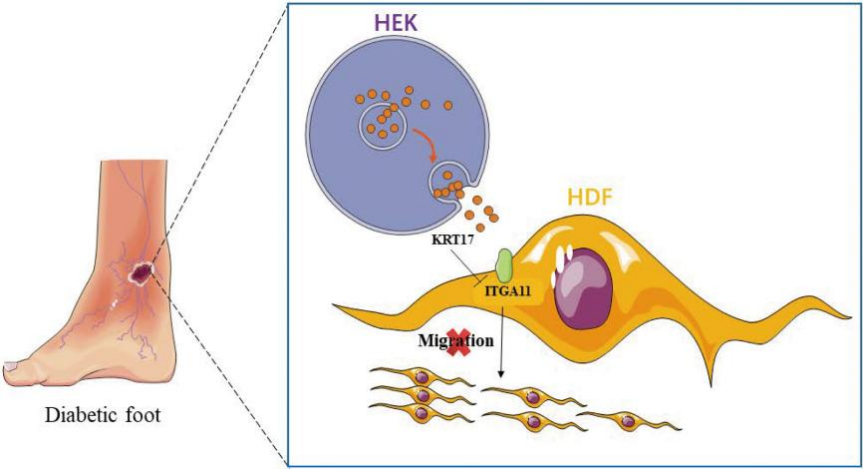
Experimental mechanism diagram.

To investigate the gene expression changes and interactions of skin cells under high conditions, we established 3 major skin cell models (HEK, HDF, and HDMEC) stimulated by high glucose in the preliminary stage and we performed RNA-seq analysis on the cell samples. In this study, we first verified the upregulation of KRT17 mRNA (mainly expressed by HEK) in 3 types of skin cells under high glucose stimulation in high glucose stimulation cell models, diabetic animal models, and skin tissues of diabetic patients. Based on the hypothesis that KRT17 may play a role in diabetic skin lesions, especially on the effect of HDF, we established an in vitro KRT17-stimulated cell model of HDF, observed changes in HDF cell proliferation and migration, and performed RNA-seq analysis. KRT17 downregulated ITGA11 expression in HDF, thereby inhibiting HDF cell migration (Fig 9). We suggest that KRT17, which is upregulated under diabetic pathological conditions, could be involved in delayed diabetic wound healing through the inhibition of HDF cell migration.

Although previous studies have demonstrated the regulatory role of KRT17 in various dermatological conditions such as congenital nail hypertrophy [23], multiple lipodystrophies [24], congenital alopecia [25], psoriasis [26], and acute and chronic wound healing [2], it is not known whether KRT17 is associated with delayed diabetic skin wound healing. In an RNA-seq analysis of oral mucosal ulcers and diabetic foot ulcer tissues, one investigator found that keratins associated with trauma activation (KRT6, KRT16, and KRT17) and keratins associated with cell differentiation (KRT1, KRT2, and KRT10) were upregulated in oral mucosal ulcer tissues to promote ulcer wound repair. However, all keratins were downregulated in diabetic foot chronic ulcer tissues, except for KRT17 [27]. The significant upregulation of KRT17 in chronic diabetic foot ulcer tissues suggests that KRT17 may be involved in the delayed healing of diabetic foot ulcers. However, another study showed significant downregulation of KRT17 in chronic nonhealing ulcers in RNA-seq analysis of healing ulcers vs chronic nonhealing venous ulcer tissues [2], suggesting that downregulation of KRT17 may impede the healing of chronic ulcers. These results suggest that KRT17 may play different functions in regulating wound healing under different pathological conditions, increasing the complexity of the role of KRT17 in regulating wound healing and requiring further in-depth studies.

Wound healing is a multistage and overlapping biological process that involves the coordinated cooperation of multiple cells. The directed migration of fibroblasts is an important factor in accelerating wound healing. Any cause of impaired fibroblast migration results in delayed wound healing. Early in wound healing, fibroblasts are recruited to migrate to the wound surface and secrete various cytokines in preparation for the next phase of healing [28, 29]. Subsequently, large numbers of fibroblasts migrate directionally to the wound surface with the support of the early extracellular matrix (ECM), where they accumulate and transform into myofibroblasts, enhancing the contractility of the wound and promoting recovery [21]. In diabetic pathological conditions, stimulation of hyperglycemia and glycosylated ECM can inhibit dermal fibroblast migration [30-32].

Significant enrichment analysis of the RNA-seq data KEGG pathway after KRT17 stimulation of HDF showed that the PI3K-AKT signaling pathway was enriched to the highest number of genes. Previous studies have reported that the PI3K-AKT signaling pathway plays an important role in regulating cell migration [33, 34]. We screened 22 differential genes in the PI3K-AKT signaling pathway and found that ITGA11, a key molecule capable of regulating cell migration, was significantly downregulated, and the downregulation of ITGA11 was verified in a cell model of KRT17-stimulated HDF at the molecular, protein, and cellular levels.

Integrins are a family of proteins that mediate cellular ECM interactions, and cell-ECM interactions are essential for fundamental biological processes, such as cell proliferation, cell differentiation, cell migration, apoptosis, morphogenesis, and organogenesis [35]. ITGA11 is a type I collagen-binding β1 integrin expressed mainly by fibroblasts [36]. Previous studies have shown that ITGA11 mediates the contraction of fibrillar collagen gels in a manner similar to ITGA2 [37], and that ITGA11 deficiency reduces the strength of granulation tissue and wounds. Further studies have revealed that ITGA11 expression is associated with myofibroblast differentiation, matrix reorganization, and collagen deposition [38-40]. Another key role of ITGA11 is the regulation of cell migration [41], especially the promotion of fibroblast migration [42-44]. Some researchers have found that ITGA11 functions by activating the PI3K-AKT signaling pathway [45]. Therefore, it is reasonable to believe that KRT17 regulates the PI3K-AKT signaling pathway by downregulating ITGA11 to inhibit HDF cell migration, which in turn is involved in delayed diabetic wound healing.

Skin stability is maintained by the self-balancing function of epidermal cells and the integrity of connective tissue, and epidermal-dermal cells can interact with each other through important signals provided by cytokines to regulate the repair of traumatized skin [46]. Interactions between keratinocytes and fibroblasts play a dominant role in the later stages of trauma healing, including the induction of fibroblast differentiation into myofibroblasts by keratinocyte paracrine secretion and promotion of fibroblast ECM secretion by keratinocytes [47]. In our study, we found that keratinocytes can regulate cell migration and collagen synthesis of HDF through KRT17, providing new insights into the mechanism of epidermal-dermal cell interactions during trauma healing.

Although our study showed that KRT17 inhibits skin fibroblast migration in vitro, the mechanism by which KRT17 acts has only been preliminarily explored and needs to be elucidated in more in-depth studies. In addition, our study lacks an animal model of diabetic skin allograft incision to further confirm and clarify the effect of KRT17 on diabetic wound healing, as well as the effect of KRT17 on other skin cells, such as skin keratin-forming cells, dermal microvascular endothelial cells, and skin inflammatory cells, which are also directions for our future research. The next step in this study is to construct a mouse model of diabetic skin allograft incision with intervention of KRT17 expression using KRT17-KO transgenic mice and/or murine tail vein injection of KRT17 neutralizing antibody and formulation of KRT17-siRNA cream to evaluate the healing of diabetic mouse skin wounds after intervention of KRT17 expression and to provide new ideas for clinical diabetic ulcer wounds.

## Data Availability

All raw data and materials are available upon reasonable request from the corresponding authors.

## Abbreviations

DEG: differentially expressed gene
ELISA: enzyme-linked immunosorbent assay
HDF: human dermal fibroblast
HDMEC: human dermal microvascular endothelial cell
HaCaT: human immortalized keratinocyte
HEK: human epidermal keratinocyte
ITGA11: integrin alpha-11
KRT17: keratin 17
PBS: phosphate-buffered saline
qPCR: quantitative polymerase chain reaction
STZ: streptozotocin
